# Secretome Profiling Reveals Virulence-Associated Proteins of *Fusarium proliferatum* during Interaction with Banana Fruit

**DOI:** 10.3390/biom9060246

**Published:** 2019-06-23

**Authors:** Taotao Li, Yu Wu, Yong Wang, Haiyan Gao, Vijai Kumar Gupta, Xuewu Duan, Hongxia Qu, Yueming Jiang

**Affiliations:** 1 Key Laboratory of Plant Resource Conservation and Sustainable Utilization, Guangdong Provincial Key Laboratory of Applied Botany, South China Botanical Garden, Chinese Academy of Sciences, Guangzhou 510650, China; taotaoli@scbg.ac.cn (T.L.); 18255137100@163.com (Y.W.); xwduan@scbg.ac.cn (X.D.); q-hxia@scbg.ac.cn (H.Q.); 2 University of Chinese Academy of Sciences, Beijing 100039, China; 3 Zhongshan Entry-Exit Inspection and Quarantine Bureau, Zhongshan 528403, China; wrone@163.com; 4 Key Laboratory of Post-Harvest Handling of Fruits, Ministry of Agriculture, Zhejiang Academy of Agricultural Sciences, Hangzhou 310021, China; spsghy@163.com; 5 Department of Chemistry and Biotechnology, ERA Chair of Green Chemistry, Tallinn University of Technology, 12618 Tallinn, Estonia

**Keywords:** secreted proteins, pathogenicity, fungi, plant host, gene expression, virulence

## Abstract

Secreted proteins are vital for the pathogenicity of many fungi through manipulating their hosts for efficient colonization. *Fusarium proliferatum* is a phytopathogenic fungus infecting many crops, vegetables, and fruit, including banana fruit. To access the proteins involved in pathogen–host interaction, we used label-free quantitative proteomics technology to comparatively analyze the secretomes of *F. proliferatum* cultured with and without banana peel in Czapek’s broth medium. By analyzing the secretomes of *F. proliferatum*, we have identified 105 proteins with 40 exclusively secreted and 65 increased in abundance in response to a banana peel. These proteins were involved in the promotion of invasion of banana fruit, and they were mainly categorized into virulence factors, cell wall degradation, metabolic process, response to stress, regulation, and another unknown biological process. The expressions of corresponding genes confirmed the existence of these secreted proteins in the banana peel. Furthermore, expression pattern suggested variable roles for these genes at different infection stages. This study expanded the current database of *F. proliferatum* secreted proteins which might be involved in the infection strategy of this fungus. Additionally, this study warranted the further attention of some secreted proteins that might initiate infection of *F. proliferatum* on banana fruit.

## 1. Introduction

The “secretome” is well-known as the collection of proteins that are delivered into the extracellular milieu via the secretory pathway [1]. Although fungal secretomes vary in composition and responsiveness between species [2], some secreted proteins, such as enzymes involved in carbohydrate degradation (the so-called cell wall-degrading enzymes or CWDEs), protein degradation, along with housekeeping enzymes, allergens, and proteins of unknown function are typically identified [3]. Previous research also showed that some secretory proteins in pathogens could manipulate and/or destroy host cells with special signatures [4]. For example, the secreted proteins in plant-associated fungi were reported to play important roles in plant and fungi symbiosis or fungal pathogenicity [5]. Therefore, the secreted proteins of fungi have recently attracted the attention of researchers due to their potentially important roles in establishing pathogenicity [3]. Furthermore, analyzing the secretomes of a phytopathogen during a dynamic interaction with its host would be a direct and idealistic approach to obtain a comprehensive snapshot of its pathogenic mechanism [6].

Targeted proteomic approaches were used to identify candidate effectors from the secreted proteins during infection [7,8]. Yang et al. also identified *F. graminearum* secreted proteins involved in the interaction with barley and wheat using gel-based proteomic analysis [9]. However, some proteomics studies of secreted proteins only identified a few fungal proteins due to their low abundance in the host [10], which is the major challenges of studying fungal secreted proteins in planta. Tian et al. also pointed out that the development of new omics techniques to unravel virulence factors in the pathogen is the primary challenge that we face in the molecular characterization of fruit-pathogen interactions [11]. Label-free quantification (LFQ) is an accurate and robust proteome-wide quantitative proteomics technology developed recently [12,13]. Furthermore, MaxLFQ was also an efficient method for the systematic detection of secreted proteins [14].

*Fusarium proliferatum* has been reported as a pathogen of many crops, fruit, and vegetables worldwide [15,16]. *F. proliferatum* has previously been isolated from the decayed banana peel [17] and Kamel et al. reported that *F. proliferatum* was involved in banana crown rot [18]. Additionally, *F. proliferatum* also produce mycotoxin such as fumonisins which are harmful to human and animals [19]. Given the significant loss in the quality of fruit and potential harm caused by *F. proliferatum*, elucidation of this fungus infection mechanism in respect of secreted proteins is essential. Previous research investigated the effect of different media on the secretome of *F. proliferatum* [20,21]. These studies may not closely mimic the nutritional situation or infection environment of the fungus in planta. Moreover, due to the limitation of gel-based proteomic technology, very few effector proteins have been functionally characterized in *F. proliferatum*.

Secretomes may be obtained from cultures growing in vitro amended with plant extracts that attempt to mimic more closely conditions that may be encountered in vivo. Importantly, Zhang et al. identified effector proteins from *V. dahliae* strain V592 cultured in CD medium supplemented with or without cotton roots using mass spectrometry (MS) [22]. Hence, *F. proliferatum* grown with plant host can be expected to induce the production of secreted proteins which would most probably be important during interaction with the host. Also, we reasoned that the altered secretomes caused by plant host might narrow down the list of secreted proteins with potential roles in establishing pathogenicity of *F. proliferatum* on its host. We proposed that secreted proteins that were induced in response to banana peel were necessary to understand early events in host–pathogen interactions.

In the present study, we identified secreted proteins from *F. proliferatum* cultured with (+BP) and without banana peel (-BP) using label-free quantitative proteomics technology. Considering most effector genes are host-induced [23], in this study, we mainly focused on the proteins that were exclusively secreted and increased in abundance in response to the banana peel. We, therefore, expected to identify proteins specifically secreted in response to the banana peel and have discussed links between the specificity of key proteins of the secretome with the corresponding virulence phenotype. Finally, our analysis of candidate virulence protein can provide valuable knowledge that will eventually contribute to the design of new and effective management to control *Fusarium* related disease. 

## 2. Materials and Methods

### 2.1. Strain and Culture Condition

The *F. proliferatum* strain ZYF was stored in 30% glycerol at −80 °C. Before use, the strain was dropped onto a potato dextrose agar (PDA, Oxoid, Basingstoke, Hampshire, England) plate and then incubated at 28 °C in the dark for 7 days. To collect fungal culture filtrate for MS, fungal agar plugs from 7-d-old plates were incubated in 200 mL Czapek’s broth (CB) medium (3.0 g/L NaNO_3_, 1.0 g/L K_2_HPO_4_, 0.5 g/L MgSO_4_·7H_2_O, 0.5 g/L KCl, 0.01 g/L FeSO4 and 30 g/L sucrose) sterilized previously at 121 °C for 20 min. Before incubation of *F. proliferatum*, the CB medium were cooled at room temperature and supplemented with and without approximately 5 g surface-sterilized banana peel (+BP/-BP). After incubation at 28 °C with shaking at 200 rpm for 3 days, the banana peel was removed, and the filtrate was collected for protein and fumonisin extraction. Three independent biological replications were carried out in this study.

### 2.2. Protein Extraction and Sample Preparation for a Label-Free Experiment

The filtrate was ultra-filtrated in 3KD ultrafiltration device, with 14,000 g for 10 min. The solution was filtered through a 0.22 µm Millipore membrane filter before being mixed with SDT solution (4% SDS, 100 mM DTT, 150 mM Tris-HCl, pH 8.0). After grounded with liquid nitrogen, five volumes of 10% TCA in acetone were added. Then the sample was vortexed and stored at −20 °C for at least 4 h. The pellets were collected after centrifugation at 6 000 g and 4 °C for 40 min and then washed with cold acetone for three times. The pellets were dried, and then the protein powder was re-suspended in 30 volumes of SDT lysis buffer (4% SDS, 100 mM DTT, 150 mM Tris-HCl, pH 8.0) and boiled for 5 min. The samples were further ultra-sonicated and boiled again for another 15 min. Undissolved cellular debris was removed by centrifugation at 14,000 g for 40 min. The supernatant was filtered through a 0.22 µm Millipore membrane filter and quantified with a Bicinchoninic acid (BCA) Protein Assay Kit (Bio-Rad, USA).

### 2.3. Protein Digestion

Digestion of protein (250 µg for each sample) was performed according to the filter aided sample preparation (FASP) procedure described by Wisniewski et al. [24]. Briefly, the detergent, DTT and other low-molecular-weight components were removed using 200 µL Urea (UA) buffer (8 M Urea, 150 mM Tris-HCl, pH 8.0) by repeated ultrafiltration (Microcon units, 30 kD) facilitated by centrifugation. Then 100 µL 0.05 M iodoacetamide in UA buffer was added to block reduced cysteine residues and the samples were incubated for 20 min in dark. The filter was washed with 100 µL UA buffer three times and then 100 µL 25 mM NH_4_HCO_3_ twice. Finally, the protein suspension was digested with 3 µg trypsin (Promega) in 40 µL of 25 mM NH_4_HCO_3_ overnight at 37 °C, and the resulting peptides were collected as a filtrate. The peptide content was estimated by UV light spectral density at 280 nm using an extinction coefficient of 1.1 of 0.1% (g/L) solution that was calculated based on the frequency of tryptophan and tyrosine in vertebrate proteins.

### 2.4. Liquid Chromatography (LC)-Electrospray Ionization (ESI) Tandem MS (MS/MS) Analysis by Q Exactive

The peptide of each sample was desalted on C18 Cartridges (Empore™ SPE Cartridges C18 (standard density), bed ID 7 mm, volume 3 mL, Sigma), then concentrated by vacuum centrifugation and reconstituted in 40 µL of 0.1% (*v/v*) trifluoroacetic acid. MS experiments were performed on a Q Exactive mass spectrometer that was coupled to Easy nLC (Proxeon Biosystems, now Thermo Fisher Scientific). 5 µg peptide was loaded onto a the C18-reversed phase column (Thermo Scientific Easy Column, 10 cm long, 75 µm inner diameter, 3 µm resin) in buffer A (2% acetonitrile and 0.1% Formic acid) and separated with a linear gradient of buffer B (80% acetonitrile and 0.1% Formic acid) at a flow rate of 250 nL/min controlled by IntelliFlow technology over 120 min. MS data were acquired using a data-dependent top10 method dynamically choosing the most abundant precursor ions from the survey scan (300–1800 *m*/*z*) for higher energy collision dissociation (HCD) fragmentation. Determination of the target value is based on predictive Automatic Gain Control (pAGC). Dynamic exclusion duration was 25 s. Survey scans were acquired at a resolution of 70,000 at *m*/*z* 200 and resolution for HCD spectra was set to 17,500 at *m*/*z* 200. The normalized collision energy was 30 eV and the underfill ratio, which specifies the minimum percentage of the target value likely to be reached at maximum fill time, was defined as 0.1%. The instrument was run with peptide recognition mode enabled. MS experiments were performed triply for each sample.

### 2.5. Sequence Database Searching and Data Analysis

The MS data were analyzed using MaxQuant software version 1.3.0.5. MS data were searched against the *Fusarium proliferatum* ET1 protein database (https://www.ncbi.nlm.nih.gov/genome/proteins/2434?genome_assembly_id=295197) and banana genome database (http://banana-genome-hub.southgreen.fr/home). Though there were several published genome databases of *F. proliferatum* (https://www.ncbi.nlm.nih.gov/genome/genomes/2434), only four strains had annotation information. Among them, ET1 strain had the best assembly results with fewest scaffolds. In addition, ET1 strain had more annotated genes and proteins. Hence, we chose ET1 strain for effector identification in this study. The detailed information for search parameters is shown in Table S2, Supplementary Materials. Label-free quantification was carried out in MaxQuant as previously described (Quantitative proteomics reveals subset-specific viral recognition in dendritic cells). Protein abundance was calculated based on the normalized spectral protein intensity (LFQ intensity). Proteins with LFQ intensity only in +BP sample were identified as exclusively secreted proteins in the +BP sample. Proteins with higher accumulation in +BP sample were identified as those with normalized total intensity ratios of (+BP: -BP) >2 combined with *p*-value < 0.05.

### 2.6. Bioinformatics Analysis

Secreted proteins were validated using SignalP 4.1 (http://www.cbs.dtu.dk/services/SignalP/; [25]), TargetP 1.1 (http://www.cbs.dtu.dk/services/TargetP/, [26]), SecretomeP 2.0 (http://www.cbs.dtu.dk/services/SecretomeP/, [27]) and fungal secretome database (http://fsd.snu.ac.kr/, [4]) and Fungal Secretome KnowledgeBase (http://bioinformatics.ysu.edu/secretomes/fungi.php, [28]). Transmembrane domains analysis was conducted using TMHMM (http://www.cbs.dtu.dk/services/TMHMM/, [29]) to exclude proteins predicted to target membranes. We just retained the proteins with no transmembrane and the whole sequence is labelled as outside. Potential virulence-related proteins were speculated according to Pathogen–Host Interactions database (PHI-base) (http://www.phi-base.org/index.jsp). Nuclear localization signal (NLS) sequence was predicted by cNLS Mapper (http://nls-mapper.iab.keio.ac.jp/cgi-bin/NLS_Mapper_form.cgi).

### 2.7. Fumonisin Production Analysis

After collection of 3-day-old culture (50 mL), Fumonisin B (FB) analysis was conducted according to previous research [19].

### 2.8. Fusarium Proliferatum Inoculations

The spore solution of *F. proliferatum* was diluted to 1 × 10^6^ spores/mL for fruit inoculation. The green mature banana fruit were washed with sterile water, and then inoculated with 15 µL of aqueous conidia suspension described above. The inoculated fruit were stored at 22 °C and 85% relative humidity (RH). The peel tissues of banana fruit with and without *F. proliferatum* infection were collected at 4 days and 10 days after inoculation immediately ground into a powder with liquid nitrogen and stored at −80 °C for further analysis. Three biological replicates were conducted.

### 2.9. Quantitative Real-Time PCR (qRT-PCR) Analysis

RNA was extracted from 5 g of ground banana peel according to Shan et al. [30] followed by the removal of genomic DNA and cDNA synthesis. Specific primers for *F. proliferatum* genes were designed using Primer Premier 6 and shown in Table S3, Supplementary Materials. *F. proliferatum* Actin served as a reference gene for the quantification of gene expression. qRT-PCR was conducted according to Li et al. [19]. Three biological replicates were performed.

### 2.10. Statistics

All data were the average value of three replicate assays ± standard errors. Data for each sample were statistically analyzed using the Student’s t-test (*p* < 0.05).

## 3. Results

### 3.1. Identification of Secreted Proteins in Response to the Banana Peel

To investigate the possible effectors which are secreted extracellularly during the interaction with the host, *F. proliferatum* was cultured in CB medium supplemented with or without banana peel (+BP/-BP). Three days after culture, the culture filtrate was collected and concentrated for Liquid Chromatography-Electrospray Ionization Tandem MS (LC-ESI-MS/MS) analysis. We identified 1324 non-redundant proteins with 340696 total spectra and 7799 peptides. The details for the identification and quantitation are shown in Figure S1, Supplementary Materials. After the bioinformatics analysis, 108 proteins were identified as secreted proteins. Three banana proteins, mitochondrial phosphate transport protein, GTP-binding protein SAR1and 40S ribosomal protein S27-2, were found in the culture. All other 105 identified proteins originated from the fungus, and the detailed information as shown in Table S1, Supplementary Materials. Among them, 40 proteins were only identified in the +BP sample (Table 1) while 65 exhibited greater intensity in +BP sample (Table 2). In total, 70 fungal proteins had an N-terminal signal peptide sequence predicted by SignalP or TargetP, indicating that they are secreted proteins. Moreover, SecretomeP suggested that 35 proteins might be secreted in a nonclassical way (Table 1 and Table 2). The Basic Local Alignment Search (BLAST) comparison with the database of fungal secreted proteins enabled further confirmation of these secreted proteins.

**Figure 1:**
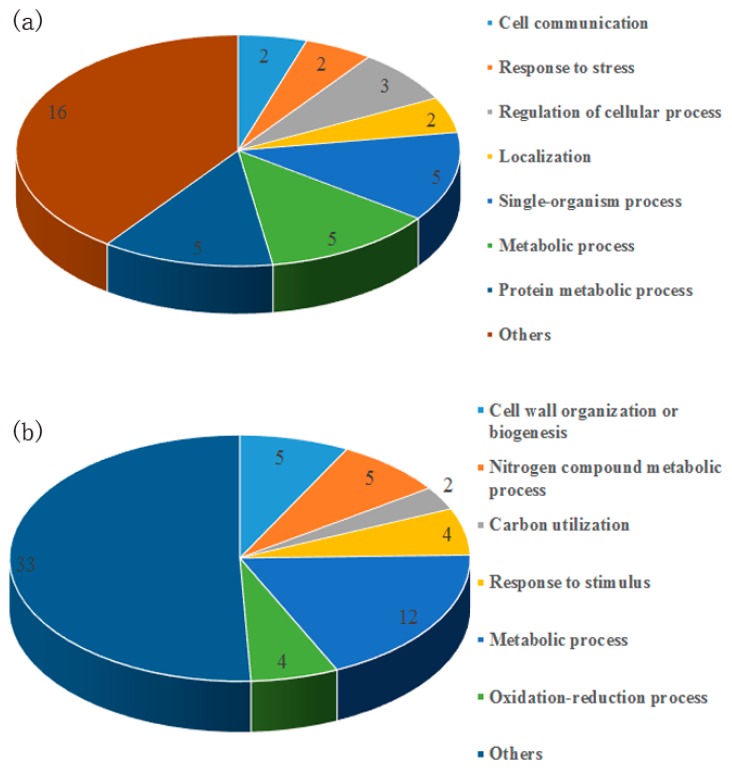
Classification of identified secreted proteins into functional categories using Blast2Go. (**a**) Proteins exclusively secreted in the +BP sample; (**b**) Proteins with higher accumulation in the +BP sample.

According to Blast2GO analysis, the secreted proteins only identified in the +BP sample were mainly categorized into the single-organism process, protein metabolic process, metabolic process, regulation of cellular process, and others (Figure 1a). Meanwhile, the secreted proteins with higher accumulation in the +BP sample were mainly categorized into metabolic process, nitrogen compound metabolic process, cell wall organization or biogenesis, response to a stimulus, oxidation-reduction process, and others (Figure 1b). The predicted secretome of *F. proliferatum* primarily contained proteins of unknown function and uncharacterized proteins (Figure 1, Table 1 and Table 2).

Of particular interest are proteins that could be acting as virulence factors. Hence, we identified protein sequences against Pathogen–Host Interactions database (PHI-base) that is an invaluable resource in the discovery of virulence and effector genes (The Pathogen–Host Interactions database (PHI-base, http://www.phi-base.org/index.jsp): additions and future developments). With few exceptions, nearly all the secreted proteins got positive results in PHI-base, and the corresponding PHI ID is shown in Table S1, Supplementary Materials. These candidate virulence proteins merit further investigation.

### 3.2. Gene Expression in Planta

**Figure 2:**
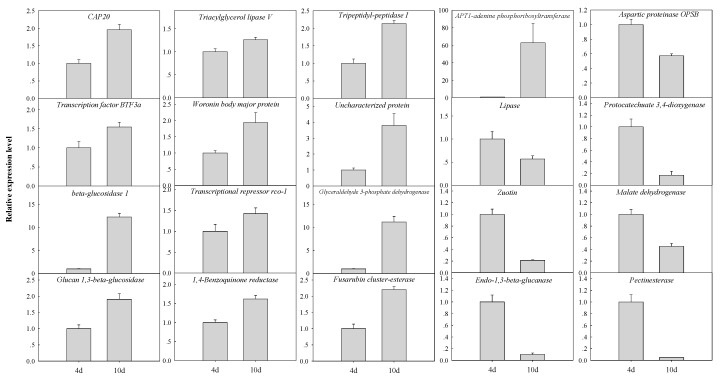
Fungal gene expression analysis in infected banana fruit by quantitative reverse transcriptase-polymerase chain reaction (qRT-PCR) at 4 and 10 days post-inoculation (dpi). The expression levels of each gene were expressed as a ratio relative to the day 4, which was set at 1. The descriptions of each gene were the same, as shown in Table 1 and Table 2.

To determine whether genes encoding the secreted proteins identified in liquid media were also expressed in planta, banana fruit were inoculated with the fungus. At 4 dpi, *F. proliferatum* infected banana fruit successfully, and at 10 dpi, disease spot and mycelium was evidently visible in inoculated banana fruit (Figure S2, Supplementary Materials). The expression patterns of 20 fungal genes encoding selected secreted proteins were analyzed in infected banana fruit at 4 and 10 dpi using quantitative real-time PCR (qRT-PCR) (Figure 2). The genes were chosen to represent a variety of functions, i.e., virulence-related (*CAP20*), protein metabolism (*aspartic proteinase OPSB*), degradation of host cell walls (*beta-glucosidase, glucan 1,3-beta-glucosidase, endo-1,3-beta-glucanase, pectinesterase*), proteins with lipase function (*lipase, triacylglycerol lipase V*), metabolism (*malate dehydrogenase, glyceraldehyde 3-phosphate dehydrogenase, APT1-adenine phosphoribosyltransferase, protocatechuate 3,4-dioxygenase, 1,4-Benzoquinone reductase, fusarubin cluster-esterase*) and regulation function (*transcription factor BTF3a, transcriptional repressor rco-1, zuotin*), and others (*woronin body major protein, tripeptidyl-peptidase I, uncharacterized protein*). Among these genes, there were 12 genes (*CAP20, aspartic proteinase OPSB, lipase, triacylglycerol lipase V, APT1-adenine phosphoribosyltransferase, protocatechuate 3,4-dioxygenase, transcription factor BTF3a, transcriptional repressor rco-1, zuotin, woronin body major protein, tripeptidyl-peptidase I, uncharacterized protein*) that encode proteins exclusively secreted in +BP sample while 8 genes (*beta-glucosidase, glucan 1,3-beta-glucosidase, endo-1,3-beta-glucanase, pectinesterase, malate dehydrogenase, glyceraldehyde 3-phosphate dehydrogenase, 1,4-Benzoquinone reductase, fusarubin cluster-esterase*) that encode proteins with higher abundance in +BP sample. As shown in Figure 2, the expression of all selected genes was detected in infected fruit, but not in uninfected controls (data not shown). Additionally, more genes were up-regulated in the late infection stage. The detection of these genes further suggested the important role of these secreted proteins in the infection of *F. proliferatum* on banana fruit.

### 3.3. Fumonisin Production

**Figure 3:**
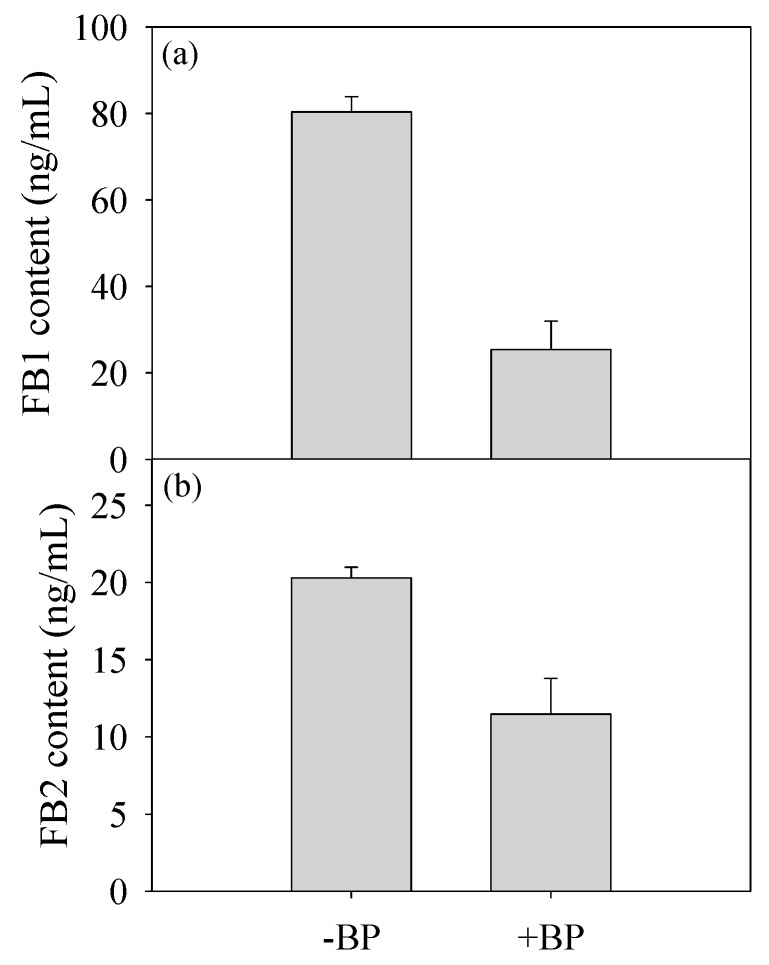
Concentration of the mycotoxin contents. (**a**) Fumonisin B1 (FB1); (**b**) fumonisin B2 (FB2) in response to banana peel, determined in triplicate.

To further analyze whether banana peel amendment affected the fumonisin production, a 3-day culture was used for the analysis of the fumonisin production. As shown in Figure 3, the concentrations of FB1 and FB2 in +BP sample were much lower than those in –BP sample. 

## 4. Discussion

Many plant pathogens can modulate their secretomes in response to their plant hosts [7,31,32,33]. In particular, fungal pathogens of plants adapt to the host environment through the secretion of proteins and other molecules to facilitate nutrient acquisition and overcome the immune response. Proteomics techniques have been widely used to identify many extracellular proteins [34,35]. *F. proliferatum* causes great loss of crops, fruit, and vegetables worldwide. This study was aimed to investigate the infection mechanism of *F. proliferatum* on banana fruit based on the secretome change in response to banana using label-free quantitative proteomic technology.

The exploration of phytopathogen secretomes has been mainly achieved through functional proteomics analyses. Due to the challenge to identify fungal proteins in planta, Yang et al. identified the secreted proteome of *F. graminearum* cultured with barley or wheat flour to represent the natural hosts of the fungus [9]. In this study, to further mimic the natural infection environment of the fungus in planta, *F. proliferatum* was cultured in medium supplemented with or without banana peel (+BP/-BP). The banana peel closely represents the natural hosts of the fungus in contrast with previous studies of the *F. proliferatum* in vitro secretome [20,21]. This approach, combined with label-free quantitate proteomic technology, which is ideally suited for a gel-free shotgun analysis [36], can identify many fungal secreted proteins. In the present study, we identified 40 highly abundant proteins only observed in the secretome of +BP sample (Table 1). Meanwhile, we also identified 65 proteins observed with up-regulation in the secretome of +BP sample (Table 2). The detection of all the selected transcripts in banana peel (Figure 2) has confirmed the relevance of the approach for the identification of fungal proteins involved in the interaction with the host. Therefore, this study increased the value of the information gained from the analysis of *F. proliferatum* secretome in vitro and provided an ideal model to understand plant–pathogen interactions.

Secretory pathways in fungi are complex and involved with the classical secretory pathway and alternate routes for protein trafficking. For the classical secretory pathway, signal peptides are added to target translated proteins outside the cell or to an organelle. In contrast, proteins with no signal peptides can be secreted via a nonclassical secretory pathway (on-conventional protein secretion in yeast). Proteins can also be secreted through other unconventional pathways, such as secretory lysosomes, exocytosis, microvesicles or ATP-binding cassette transporters [37]. Indeed, 35 of the proteins identified in the present study, including GAPDH, malate dehydrogenase and FBA1-fructose-bisphosphate aldolase, and 26S proteasome regulatory subunit RPN8, etc. were predicted to be secreted in a nonclassical way using SecretomeP (Table 1 and Table 2). Previous research also showed that several housekeeping enzymes, including GAPDH, can be secreted extracellularly by pathogens and serve as virulence factors [38,39]. Usually, ribosomal proteins in the secretome would likely be related to contaminants of the secretome when cell breakage occurs [3]. However, in the present study, we identified one 60S ribosomal protein L8 and one ribosomal protein L7a. After a BLASTp search of these protein sequences against Fungal Secretome Database (http://fsd.riceblast.snu.ac.kr/index.php?a=view), we indeed got positive results with 60S ribosomal protein from *Histoplasma capsulatum* (87.4% identity) and 60S ribosomal protein L8 from *Blastomyces dermatitidis* (82.06% identity), respectively. Mulugeta et al. also found ribosomal proteins in the secretome of *Staphylococcus carnosus*, and they pointed that the appearance of these proteins referred to as “nonclassical protein excretion” [40], which has also been observed in other pathogens. Importantly, Lu et al. showed that ribosomal proteins play essential roles in *C. albicans* virulence [41]. Thus, the exact role of ribosomal proteins in the infection ability of *F. proliferatum* still need further investigation.

PHI-base is a web-accessible database that catalogues experimentally verified pathogenicity, virulence and effector genes from bacterial, fungal and protist pathogens [42]. Analysis of secreted proteins against PHI-base indicated that proteins belonging to pathogenicity-related class were abundant in secretome in response to banana peel (Table S1, Supplementary Materials). All of them were either exclusively secreted or increased to several folds in abundance in response to banana peel tissue (Table 1 and Table 2). Highly represented proteins in this class included CAP20-virulence factor, lipase, SnodProt1, associated with virulence of fungus on plant host [43,44,45,46]. Other proteins previously associated with pathogenicity of fungus included CWDEs (beta-glucosidase, glucan 1,3-beta-glucosidase, endo-1,3-beta-glucanase and pectinesterase), proteases (aspartic proteinase OPSB, subtilisin-like serine protease), endochitinase 2, all of which have well documented roles in plant pathology as enzymes that either degrade the cell wall to permit access to the host or neutralize the host defenses [47,48,49]. Additionally, the results of the in planta expression profiling indicated that these genes had different expression patterns at different infection stages (Figure 2). For example, genes encoding glucan endo-1, 3-beta-glucanase, pectinesterase and aspartic proteinase OPSB showed higher expression level at early infection stage, suggesting they might mainly aid *F. proliferatum* at early time points in penetration and colonization of banana peel. Barnabas et al. also reported that fungus could secrete and regulate the expression of some secretory proteins at distinct stages of infection [6].

Proteins involved in nutrition absorption were also present and included SUC2-invertase, acid phosphatases and they were both involved the successful colonies of the fungus through deriving nutrients from their host [50]. Some proteins involved in the oxidation-reduction process, nitrogen compound metabolic process, carbon use process was also identified in the secretomes in the present study (Figure 1, Table 1 and Table 2). As reported by previous research, nitrogen metabolism, carbohydrate metabolism and oxidoreductase reactions are beneficial for the fungus to cope up and adapt to different nutritional environments during infection stages [6].

The analysis of the secretome also revealed 13 proteins with NLS (Table 1 and Table 2). NLS was reported to predict the in planta localizations of fungal proteins [51]. Previous research has reported that some various bacterial, oomycete and fungal effectors targeted in host nucleus [52,53,54]. However, no *F. proliferatum* effectors have been found to target the host nucleus and trigger plant immunity. Zhang et al. also identified a novel secreted protein VdSCP7 that targets the plant nucleus and modulate plant immunity through analyzing the secretomes of *V. dahlia* [22]. Indeed, we identified two transcription regulation factor (transcription factor BTF3a and transcriptional repressor rco-1), one zuotin as well as one rAsp f 9 allergens, which were nuclear localized effector (Table 1 and Table 2). Rampitsch et al. also identified allergen proteins in the secretomes of *F. graminearum* [3]. Furthermore, an allergen Asp F4-like protein was the causal agent of stem rot in corn [55]. So far, the role of a transcription factor in pathogenesis mainly focused on fungus itself [56,57]. No transcription factor has directly been secreted extracellular to regulate plant immunity. The secreted proteome in this study could anticipate their role in pathogen–host interactions. Our present results cannot shed light on the function of these proteins, but these proteins are indeed widely secreted by *F. proliferatum* in response to the plant host. Therefore, these are good candidates for further analysis of their exact role in pathogenicity.

Mycotoxin is also an important secreted molecule of fungus. Fumonisins are a group of mycotoxins mainly derived from *Fusarium*, Liseola section including *F. proliferatum* [58]. Whether the production of fumonisin facilitates the pathogenesis of fungus remains an open issue [59]. Some research indicated that fumonisins appeared to be involved in disease development [60], while other research indicated that deletion of fumonisin production has no effect on the aggressiveness of fungus [61]. In this study, lower fumonisins (FB1 and FB2) content were identified in +CB culture (Figure 3). Our results suggested that the initiation of infection might not be caused by FB production but other pathogenicity factors. Desjardins et al. also indicated that the role of fumonisins depends on complex environmental and genetic contexts in host–pathogen interaction [62].

## 5. Conclusions

Our current results provided a comprehensive, comparable proteomic analysis of the secreted proteins of *F. proliferatum* induced by the interaction with the banana host. Our results indicated that *F. proliferatum* could modulate itself with a set of extracellular proteins that prepares it for encountering and infection of the host plant. Our data suggested a subset of the secreted proteins whose presence might be required to initiate infection of *F. proliferatum* and provided a foundation for future investigation of virulence factors. Gene expression in planta confirmed their secretion and variable roles in different infection stages. In sum, this study provided valuable insight into secretory capacity and pathogenicity of *F. proliferatum* as well as the molecular interactions between fungi and the plant host.

## Figures and Tables

**Table 1:** Summary of the secreted proteins of *F. proliferatum* only identified in the +BP sample. Mr: Molecular weight. P: positive results from SignalP; S: positive results from SecretomeP; T: positive results from TargetP.

Protein IDs	Protein Description	Unique Peptides	Mr (kDa)	Signal	NLS Sequence
CZR33670.1	APT1-adenine phosphoribosyltransferase	1	23.3	P	
CZR34854.1	uncharacterized protein FPRO_01025	4	96.7	P	
CZR34878.1	uncharacterized protein FPRO_01001	2	77.3	P	
CZR35312.1	uncharacterized protein FPRO_00565	2	24.0	S	
CZR37023.1	uncharacterized protein FPRO_02717	2	26.4	S	LFRTIASVAFLACASNVAAEPQPYKLVKAP
CZR37404.1	uncharacterized protein FPRO_02336	1	19.7	S	
CZR37761.1	uncharacterized protein FPRO_07048	5	20.8	S	
CZR37893.1	uncharacterized protein FPRO_06916	3	9.5	S	RSILHHCGKHASWDHAKSECVCHDSGKVYTKKHH
CZR37952.1	uncharacterized protein FPRO_06857	2	25.8	T	
CZR38705.1	CAP20-virulence factor	6	20.3	P	
CZR39121.1	uncharacterized protein FPRO_05687	1	26. 3	S	
CZR39201.1	uncharacterized protein FPRO_05607	1	39.0	S	
CZR39716.1	H+-transporting ATPase	2	85.7	P	
CZR39800.1	60S ribosomal protein L8	3	27.6	P	
CZR39939.1	ATP-dependent RNA helicase DED1	1	71.8	P	
CZR40877.1	related to SYP1 Protein with a potential role in actin cytoskeletal organization	3	96.3	P	
CZR41198.1	related to aspartic proteinase OPSB	5	49.9	S	RRRLRKRDGTIEIGIDNEQSLYFLNASLGTPP
CZR41331.1	26S proteasome regulatory subunit RPN8	2	38.3	P	
CZR41485.1	GTP-binding protein ypt1	4	22.4	P	
CZR42158.1	related to ochre suppressor tyr-tRNA	2	18.1	P	RNEVIRCLREHDRRPLDCWQEVENFKAEVKKLEKSW
CZR42368.1	transcription factor BTF3a	4	17.0	P	PRRKVKRAPARSGADDKKLQLALKKLNT
CZR42437.1	woronin body major protein precursor	3	71.0	P	
CZR42601.1	uncharacterized protein FPRO_09904	2	15.3	S	SKRQIVWPAYTDKQVQSGKVVKPD
CZR43413.1	related to lactonohydrolase	1	35.6	P	
CZR43528.1	related to 2‘-hydroxyisoflavone reductase	2	32.1	P	
CZR44148.1	potassium channel beta subunit protein	3	39.5	P	
CZR45692.1	related to tripeptidyl-peptidase I	6	64.9	T	
CZR46340.1	related to protocatechuate 3,4-dioxygenase beta subunit	1	39.8	S	
CZR46385.1	related to oxidoreductase related to nitroreductase	3	22.6	P	
CZR46670.1	uncharacterized protein FPRO_12120	1	9.7	P	
CZR46904.1	cytochrome-c oxidase chain IV precursor	3	27.0	P	
CZR46998.1	related to tripeptidyl-peptidase I	6	64.9	S	
CZR47100.1	transcriptional repressor rco-1	2	66.1	P	LDRTIKMWELSAPRQGNQPGPKGGKCVKT
CZR47653.1	related to acetylxylan esterase precursor	2	30.8	T	
CZR47873.1	uncharacterized protein FPRO_13540	1	35.5	S	
CZR48663.1	related to toxD protein	5	38.3	P	
CZR48742.1	lipase precursor	6	47.5	S	
CZR48857.1	related to triacylglycerol lipase V precursor	4	57.3	S	
CZR49188.1	zuotin	1	50.4	P	ENRDQKRHQERKNTNARKKKKAD
CZR49277.1	uncharacterized protein FPRO_08983	4	19.9	S	

**Table 2:** Summary of the secreted proteins of *F. proliferatum* identified with higher abundances in the +BP sample. Mr: Molecular weight. The ratio (+BP/−BP) was calculated according to the normalized spectral protein intensity (LFQ intensity) ratios of (+BP: −BP).

Protein IDs	Protein Description	Unique Peptides	Mr. (kDa)	Signal	Ratio (+BP/-BP)	NLS Sequence
CZR47323.1	uncharacterized protein FPRO_08697	6	33.5	S	14.6	
CZR45923.1	related to beta-glucosidase 1 precursor	28	83.4	S	16.3	
CZR44287.1	uncharacterized protein FPRO_14048	3	23.2	S	8.6	
CZR49124.1	uncharacterized protein FPRO_12560	5	37.7	S	7.2	PSSKRGLIYIPNSDFPSDDKVWVQKHSDLT
CZR35354.1	probable 1,4-Benzoquinone reductase	7	21.7	P	7.7	
CZR43990.1	related to glucan 1,3-beta-glucosidase	9	33.5	S	8.4	
CZR41742.1	uncharacterized protein FPRO_11332	12	93.7	S	22.7	
CZR46647.1	uncharacterized protein FPRO_12097	5	16.1	S	5.8	DTVGKHFIPNKQLWQSKEPNAEIQRYKGPKD
CZR44412.1	related to triacylglycerol lipase V precursor	21	65.1	S	12.1	
CZR47104.1	probable malate dehydrogenase	14	34.9	P	3.1	
CZR38616.1	probable glyceraldehyde 3-phosphate dehydrogenase (ccg-7)	20	36.1	P	114.7	
CZR45734.1	related to acid phosphatase Pho610	8	48.7	S	3.0	
CZR33506.1	uncharacterized protein FPRO_01717	5	23.8	S	2.2	
CZR46837.1	probable FBA1-fructose-bisphosphate aldolase	13	39.6	P	3.7	
CZR36235.1	related to phosphatidylcholine-sterol acyltransferase precursor	4	32.6	S	7.2	
CZR47504.1	uncharacterized protein FPRO_1317	11	20.7	S	2.5	EKTWKNAHYKAGGDKAYSNRRVTCQQKQLKVP
CZR47726.1	related to glu/asp-tRNA amidotransferase subunit A	21	63.4	S	8.2	DAPSKRRLPK
CZR45085.1	probable rAsp f 9 allergen	12	41.3	S	21.9	WSKIALAGLFASAAAQTYSECNPMKKTCDP
CZR44035.1	uncharacterized protein FPRO_13841	1	28.4	S	11.1	
CZR35784.1	uncharacterized protein FPRO_00093	1	20.9	S	5.5	
CZR47211.1	related to acetylxylan esterase precursor	7	36.1	S	4.3	
CZR46230.1	uncharacterized protein FPRO_11677	8	19.1	S	12.4	
CZR36609.1	related to tyrosinase precursor	11	62.9	S	2.7	
CZR45243.1	related to lipase (lipP)	3	34.6	P	2.3	
CZR47062.1	uncharacterized protein FPRO_08436	1	14.8	S	5.1	
CZR40146.1	uncharacterized protein FPRO_05046	4	14.3	S	12.9	
CZR42211.1	ribosomal protein L7a	6	29.8	P	25.2	
CZR45507.1	cytochrome P450 55A2	16	46.9	P	2.3	
CZR37840.1	SnodProt1 precursor	3	14.6	S	8.9	
CZR38203.1	uncharacterized protein FPRO_06606	1	11.9	S	77.4	
CZR42089.1	endochitinase 2 precursor	12	88.5	P	2.8	
CZR48911.1	subtilisin-like serine protease	26	92.2	S	2.1	
CZR40535.1	related to amidase family protein	16	70.1	S	7.8	
CZR42124.1	uncharacterized protein FPRO_09425	6	19.2	S	3.4	
CZR43152.1	related to sporulation-specific gene SPS2	13	42.3	S	2.4	
CZR40180.1	uncharacterized protein FPRO_05080	2	14.8	S	6.5	
CZR42998.1	uncharacterized protein FPRO_08086	4	16.8	S	8.9	
CZR38140.1	uncharacterized protein FPRO_06669	4	32.1	S	5.8	
CZR38273.1	fusarubin cluster-esterase	12	41.2	S	5.7	
CZR36329.1	uncharacterized protein FPRO_03411	1	23.4	S	11.0	
CZR47769.1	phosphoglycerate kinase	31	44.7	P	2.1	
CZR35108.1	uncharacterized protein FPRO_00770	6	21.5	S	7.2	
CZR40410.1	uncharacterized protein FPRO_05310	3	18.4	P	11.1	
CZR38701.1	uncharacterized protein FPRO_06108	3	30.9	S	6.7	
CZR44031.1	uncharacterized protein FPRO_13838	2	79.7	S	3.9	
CZR48068.1	uncharacterized protein FPRO_12678	5	32.5	S	3.9	
CZR38172.1	related to SUC2-invertase (sucrose hydrolyzing enzyme)	10	60.2	S	2.9	
CZR49275.1	uncharacterized protein FPRO_08985	4	28.0	S	6.2	
CZR49368.1	related to BNR/Asp-box repeat domain protein	4	41.3	S	3.9	
CZR34777.1	related to extracellular matrix protein precursor	4	21.9	S	3.5	
CZR34851.1	uncharacterized protein FPRO_01028	5	15.3	S	3.8	
CZR36412.1	related to myosin heavy chain	18	133.6	P	8.3	
CZR34562.1	probable NHP6B-nonhistone chromosomal protein	2	11.5	P	7.6	
CZR44986.1	related to endo-1,3-beta-glucanase	4	33.1	S	3.3	
CZR41328.1	related to serine proteinase inhibitor IA-2	5	10.5	S	6.1	
CZR41607.1	uncharacterized protein FPRO_11196	5	22.3	S	2.5	
CZR49589.1	uncharacterized protein FPRO_15947	12	58.9	S	69.2	
CZR42484.1	related to glucan 1,3-beta-glucosidase	13	94.1	S	2.1	
CZR49007.1	uncharacterized protein FPRO_12444	3	26.1	S	2.7	
CZR41020.1	uncharacterized protein FPRO_10609	1	34.3	S	4.4	
CZR47343.1	related to acid phosphatase precursor (pH 6-optimum acid phosphatase)	3	70.1	S	8.5	
CZR34748.1	uncharacterized protein FPRO_01131	6	19.2	S	4.2	
CZR45488.1	pectinesterase precursor	7	34.9	S	2.3	
CZR39344.1	uncharacterized protein FPRO_04241	2	27.4	S	3.8	
CZR42912.1	CPC2 protein	5	35.0	P	2.2	

## Supplementary Materials

The following are available online at https://www.mdpi.com/2218-273X/9/6/246/s1, Figure S1: The evaluation results of the identification and quantitation of proteins in *F. proliferatum* cultured with and without banana peel; Figure S2: Visual picture of *Fusarium proliferatum* infection on banana fruit; Table S1: Detailed information for identified secreted proteins in this study; Table S2: Details for protein LFQ parameters; Table S3: Primers used for qRT-PCR in this study.
